# Thyroid function among women with gestational trophoblastic diseases. A cross-sectional study

**DOI:** 10.1590/1516-3180.2018.0481090519

**Published:** 2019-08-08

**Authors:** Harun Düğeroğlu, Murat Özgenoğlu

**Affiliations:** I MD. Assistant Professor, Department of Internal Medicine, Ordu Üniversitesi Tıp Fakültesi, Ordu, Turkey. Formerly at Van Yüzüncü Yıl Üniversitesi.; II MD. Specialist Doctor, Internal Medicine Clinic, Ödemiş Devlet Hastanesi, İzmir, Turkey. Formerly at Van Yüzüncü Yıl Üniversitesi.

**Keywords:** Gestational trophoblastic disease, Hydatidiform mole, Thyroid gland, Propylthiouracil

## Abstract

**BACKGROUND:**

Gestational trophoblastic diseases (GTDs) are treatable rare tumors with wide distribution. The estimated incidence of GTDs varies dramatically between different regions globally. In early pregnancy, there may be high human chorionic gonadotropin (HCG) concentrations, normal or slightly increased free T4 (fT4) and subnormal thyroid-stimulating hormone (TSH), causing hyperthyroidism ranging from subclinical to severe. Beta-HCG causes thyrotoxicosis through thyroid stimulation in patients with trophoblastic tumors.

**OBJECTIVE:**

To assess thyroid function among patients diagnosed with complete or partial hydatidiform mole, within the GTD spectrum.

**DESIGN AND SETTING:**

Cross-sectional study based on patients’ medical records at Van University Hospital, Van, Turkey.

**METHODS:**

50 patients monitored due to diagnoses of hydatidiform mole were included and were examined regarding thyroid function. Thyroid gland size and volume were measured using thyroid ultrasonography. Beta-HCG, TSH, fT4, free T3 (fT3), total T4 (TT4), total T3 (TT3), anti-thyroid peroxidase (anti-TPO), anti-thyroglobulin (anti-TG) and thyroglobulin levels were measured.

**RESULTS:**

Among these patients, 15 (30%) were diagnosed with complete hydatidiform mole and 35 (70%) with partial hydatidiform mole, according to pathology results. Those with complete hydatidiform mole were older (P = 0.003), with higher number of pregnancies (P = 0.032), lower TSH level (P = 0.011) and higher fT4 and TT4 levels (P = 0.04; P = 0.028), compared with partial hydatidiform mole patients.

**CONCLUSION:**

In hydatidiform mole patients, thyroid disease severity increases with age, parity, beta-HCG level and mole size. However, prospective multicenter studies on this topic are needed, with larger numbers of patients and closer monitoring.

## INTRODUCTION

Gestational trophoblastic diseases (GTDs) are treatable rare tumors with wide distribution. The first thyrotoxicosis case was identified in a woman with hydatidiform mole in 1955.^1^ Subsequent studies increased the numbers of cases reported.^2^^,^^3^^,^^4^ The estimated incidence of GTDs varies dramatically between different regions globally. For example, the incidence of hydatidiform mole pregnancy is 2/1000 pregnancies in Japan, while in Europe and North America it has been reported as 0.6-1/1000 pregnancies.

Tumor cells produce human chorionic gonadotropin (HCG).^5^ HCG released by hydatidiform moles has greater thyroid-stimulating activity than does HCG released from a normal placenta.^6^ The amount of HCG released is very specific and is linked to the number of live tumor cells. Women with hydatidiform mole pregnancy and choriocarcinoma may present pathologically high HCG levels. In early pregnancy, there may be high HCG concentrations, normal or slightly increased free T4 (fT4) and subnormal thyroid-stimulating hormone (TSH) level, and these may cause hyperthyroidism ranging from subclinical to severe grade.

Beta-HCG causes thyrotoxicosis through thyroid stimulation in patients with trophoblastic tumors.^2^ Beta-HCG comprises two subunits. The alpha subunit is the same as TSH, LH (luteinizing hormone) and FSH (follicle-stimulating hormone). The beta subunit is similar to the beta subunit of TSH, but is larger. Thyroid-stimulating activity of beta-HCG has been shown in mice, rats and humans.^6^^,^^7^^,^^8^

In trophoblastic diseases the spectrum of thyroid function changes varies from slight increases in fT4 and free T3 (fT3) and low TSH levels with no thyrotoxicosis symptoms, to moderate increases in fT4 and fT3 and up to increases large enough to cause clinical thyrotoxicosis or even thyroid storms.^9^ In women with hydatidiform mole pregnancy and choriocarcinoma, pathologically high HCG levels cause clear hyperthyroidism. However, thyrotoxicosis is not observed in all trophoblastic patients. Although the majority of trophoblastic tumors cause high fT4 and fT3 levels in women, some women have typical clinical findings with very few thyrotoxicosis symptoms. These include weight loss, muscle weakness, fatigue, excessive sweating, heat intolerance, tachycardia, irritability and tremors. In women with trophoblastic tumors, the thyroid gland may not grow or may display slight growth. Rarely, it may reach twice normal size. These patients do not have ophthalmopathy.^10^

Surgical removal of the hydatidiform mole rapidly ameliorates thyrotoxicosis and, if possible, surgical intervention should be performed in the early period of pregnancy. For preoperative hyperthyroidism, propylthiouracil (PTU) or methimazole may be administered.^2^

## OBJECTIVE

The aim of our study was to assess thyroid function among patients with diagnoses of complete or partial hydatidiform mole, within the gestational trophoblastic disease spectrum.

## METHODS

### Study design, date, setting and ethical concerns

Our observational study included consecutive patients who were being monitored at the gynecology and obstetrics clinic because of a diagnosis of hydatidiform mole and who underwent consultations at the internal medicine clinic regarding their thyroid function during a 20-month period. The research protocol was approved by the Research Ethics Committee of Van Yüzüncü Yıl University Faculty of Medicine, on June 17, 2011, under number 15. The patients participating in the study were informed about it and their consent was obtained.

### Participants and data collection

The patients’ ages, number of pregnancies, parity, number of surviving children, number of aborti, number of cesareans, week of pregnancy, history of goiter and previous history of hydatidiform mole pregnancy were recorded. Serum samples were studied using an Abbott Architect I4000SR device using standard methods. The patients’ initial TSH, fT4, fT3, total T4 (TT4), total T3 (TT3), beta-HCG, anti-thyroid peroxidase (anti-TPO), anti-thyroglobulin (anti-TG) and thyroglobulin (TG) levels were measured. All patients with euthyroid values were operated at the gynecology and obstetrics clinic.

The patients underwent thyroid ultrasonography at the endocrine clinic using a Siemens-Acuson 7500 device. On thyroid ultrasonography images, the patients’ thyroid size (right lobe, left lobe and isthmus) was measured. Thyroid volume was calculated using the formula: width ***x*** length ***x*** height ***x*** 0.523.^11^

### Statistical analysis

The study results were uploaded from the prepared forms to the Statistical Package for the Social Sciences for Windows software, version 22.0 (SPSS Inc, Chicago, USA), for statistical analysis. Comparison of groups was performed using the unpaired t test, and correlations between variables were analyzed using Pearson correlation analysis. Variations before and after treatment were compared using the paired-sample t test. To compare frequencies between groups, the chi-square test was used. Results were presented as the mean ± standard deviation, and P < 0.05 was accepted as statistically significant.

## RESULTS

During the study period, 50 women with a diagnosis of hydatidiform mole diagnosis were recruited. The descriptive characteristics of the patients included in the assessment are given in Table 1 and Table 2.

**Table 1.** Age and fertility characteristics of patients

	N	Min.	Max.	Mean	SD
Age (years)	50	17	54	31.08	11.31
Number of pregnancies	50	1	16	5.04	3.82
Parity	50	0	12	3.42	3.35
Number of surviving children	50	0	12	3.36	3.30
Number of aborti	50	0	4	0.61	0.99
Number of cesareans	50	0	2	0.06	0.31
Week of pregnancy at operation time	50	4	24	11.18	3.51

N = number; min. = minimum; max. = maximum; SD = standard deviation.

**Table 2.** Initial laboratory values for patients

	N	Min.	Max.	Mean	SD
TSH (uIU/ml)	50	0.01	5.32	0.72	1.08
fT4 (ng/dl)	50	0.91	4.22	1.79	0.81
fT3 (pg/ml)	50	2.22	14.71	5.38	3.32
fT3/fT4	50	0.21	0.53	0.34	0.07
TT4 (ug/dl)	50	7.62	24.01	14.53	5.51
TT3 (ng/ml)	50	0.91	7.32	2.15	1.20
Thyroglobulin (ng/ml)	50	2	262	74.18	75.59
Thyroid isthmus size (mm)	50	0	23	3.47	3.37
Total thyroid volume (mm^3^)	50	5529.11	29486.49	13515.71	6180.89
Beta-HCG (mIU/ml)	50	8159	1820573	491684.70	490615.45

N = number; min. = minimum; max. = maximum; SD = standard deviation; TSH = thyroid-stimulating hormone; fT4 = free T4; fT3 = free T3; TT4 = total T4; TT3 = total T3; Beta-HCG = beta-human chorionic gonadotropin.

The number of pregnancies among the patients ranged from 1 to 16. The mean number of pregnancies was five. Nine patients (18%) were pregnant for the first time. There were eight patients (16%) on their second pregnancy. There were seven patients on their third pregnancy (14%). There were 19 patients with six or more pregnancies (38%). There were seven patients with 10 or more pregnancies (14%).

In terms of parity (live births), our patients’ parity range was from 0 to 12. Their mean parity was 3.4. Thirteen patients (26%) had parity of one. The most common parity for our patients was one. There were 10 patients (20%) with parity of zero. There was one patient each with parity of 4, 9, 10, 11 and 12.

Thirty-three patients (66%) had no history of aborti, while 17 had had at least one abortus. The highest number of aborti was four (one patient). While 48 patients did not have histories of cesareans, there were two patients with a total of three cesareans.

Preoperatively, 33 patients (66%) did not receive antithyroid treatment, while the other 17 patients (34%) received this treatment.

According to the pathology results from the 50 hydatidiform mole patients included in the study, 15 patients (30%) were diagnosed with complete hydatidiform mole while 35 patients (70%) were diagnosed with partial hydatidiform mole. The patients were grouped according to the pathology results as presenting partial hydatidiform mole or complete hydatidiform mole. The patient group with complete mole had higher mean age (P = 0.003), higher number of pregnancies (P = 0.032), lower TSH values (P = 0.011) and higher fT4 and TT4 (P = 0.04, P = 0.028), compared with the patient group with partial mole. The thyroid volume was larger in the complete mole patients (P = 0.032). The statistical data on the complete mole and partial mole patients are shown in Table 3.

**Table 3.** Comparison of data on patients with complete and partial mole

	Pathology results	N	Mean	SD	P-value
Age (years)	Partial	35	28.03	10.39	0.003
	Complete	15	38.20	10.40	
Number of pregnancies	Partial	35	4.29	3.37	0.032
	Complete	15	6.80	4.32	
Week of pregnancy at operation time	Partial	35	11.37	3.92	0.562
	Complete	15	10.73	2.34	
TSH (uIU/ml)	Partial	35	0.91	1.21	0.011
	Complete	15	0.28	0.47	
fT4 (ng/dl)	Partial	35	1.64	0.75	0.042
	Complete	15	2.15	0.85	
fT3 (pg/ml)	Partial	35	4.85	3.001	0.283
	Complete	15	6.58	3.787	
TT4 (ug/dl)	Partial	35	12.37	4.29	0.028
	Complete	15	17.04	5.87	
TT3 (ng/ml)	Partial	35	2.04	1.26	0.422
	Complete	15	2.37	1.04	
Thyroglobulin (ng/ml)	Partial	35	59.81	70.25	0.531
	Complete	15	108.46	79.56	
Beta-HCG (mIU/ml)	Partial	35	491699.37	547934.03	1.004
	Complete	15	491650.47	336642.51	
Total thyroid volume (mm^3^)	Partial	35	12272.91	530.68	0.032
	Complete	15	16332.71	7027.52	
Thyroid isthmus size (mm)	Partial	35	3.47	3.91	0.995
	Complete	15	3.47	1.68	

N = number; SD = standard deviation; TSH = thyroid-stimulating hormone; fT4 = free T4; fT3 = free T3; TT4 = total T4; TT3 = total T3; Beta-HCG = beta-human chorionic gonadotropin.

In our study, there were 30 patients with HCG titers above 200,000 uIU/l, and 20 of them had titers above 400,000 uIU/l. Out of the 30 patients with HCG titers above 200,000 uIU/l, 13 had TSH values above 0.2 IU/l, while the remaining 17 patients had TSH levels below 0.2 IU/l. Additionally, out of the 20 patients with HCG titers above 400,000 uIU/l, five had TSH levels above 0.2 IU/l and 15 patients had TSH levels below 0.2 IU/l. Out of the 30 patients with HCG titers above 200,000 uIU/l, 17 (70%) began treatment due to clear presence of hyperthyroidism, while 15 (71%) of the 20 patients with HCG titers above 400,000 uIU/l began treatment for this reason.

Regarding the anti-TPO and anti-TG levels among the patients in this study, only three of the 50 patients (6%) were positive for anti-TPO (above 20 IU/ml) and only one patient (2%) was positive for anti-TG (above 20 IU/ml).

Among all the patients included in the study, 34 had nausea and 16 did not have nausea. Comparison between the patients with and without nausea showed that there was no significant difference between women with and without nausea regarding beta-HCG levels, thus nausea was independent of beta-HCG levels (P = 0.706). The fT4 and fT3 levels in the nausea group were identified as higher than those in the group without nausea, and this situation was significant (P = 0.042 and P = 0.023, respectively) (Table 4).

**Table 4.** Comparison of data between patients with nausea and without nausea

	Nausea status	N	Mean	SD	P-value
Age (years)	Absent	16	26.94	9.92	0.075
	Present	34	33.03	11.53	
Number of pregnancies	Absent	16	4.31	3.24	0.361
	Present	34	5.38	4.06	
Week of pregnancy at operation time	Absent	16	10.75	3.47	0.558
	Present	34	11.38	3.56	
TSH (uIU/ml)	Absent	16	1.25	1.38	0.053
	Present	34	0.48	0.81	
fT4 (ng/dl)	Absent	16	1.51	0.49	0.042
	Present	34	1.92	0.91	
fT3 (pg/ml)	Absent	16	4.13	1.77	0.023
	Present	34	5.99	3.72	
TT4 (ug/dl)	Absent	16	12.44	2.45	0.104
	Present	34	15.32	6.15	
TT3 (ng/ml)	Absent	16	1.76	0.61	0.055
	Present	34	2.34	1.37	
Thyroglobulin (ng/ml)	Absent	16	74.60	79.52	0.979
	Present	34	73.97	74.92	
Beta-HCG (mIU/ml)	Absent	16	445994.56	643480.57	0.706
	Present	34	513185.94	409520.94	

N = number; SD = standard deviation; TSH = thyroid-stimulating hormone; fT4 = free T4; fT3 = free T3; TT4 = total T4; TT3 = total T3; Beta-HCG = beta-human chorionic gonadotropin.

## DISCUSSION

In our region of Eastern Anatolia, the birth rates are high in rural areas and therefore complications linked to pregnancy are commonly observed in this population. During the 20-month study period, 50 hydatidiform mole pregnancy cases were collected. It has been reported that the risk of hydatidiform mole pregnancy is 0.6-2/1000 pregnancies.^12^

Among women with hydatidiform mole pregnancies, the risk of repetition of this condition is 10 times greater.^5^ While the risk of a second hydatidiform mole pregnancy is 1/76 for women with one previous such pregnancy, the risk of a third hydatidiform mole pregnancy is 1/7 for women with two such pregnancies.^12^ In our study, there was one patient with a second hydatidiform mole pregnancy (2%).

The risk of hydatidiform mole pregnancy is higher at the end of the reproductive period. The risk is higher especially for women over the age of 40. A study investigating 2202 hydatidiform mole pregnancies showed that the risk was significantly higher for women aged 15 and younger and for those aged 40 and older.^13^ In our study group, our youngest patient was 17 years of age, while there were 13 patients above the age of 40 years. Most patients were aged from 20 to 25 years (n = 15). To estimate whether the risk increases above the age of 40 years or not, it is necessary to know the age-specific birth rates, and we were unable to assess this information in this study (the age when women had their children).

It has been proposed that a low-protein diet, with nutrition poor in animal fats and poor in fat-soluble carotene, increases the risk of hydatidiform mole pregnancy.^14^ This risk occurs especially among individuals of low socioeconomic level. We believe that the risk of occurrence of hydatidiform mole pregnancy in our region, which has a low socioeconomic profile, is higher because of diet-linked factors.

In the group of patients with nausea, the free T4 and free T3 ­levels were identified as higher. Dopamine inhibits TSH-mediated T4 secretion.^15^ Thyroxine is not known to have a feedback control mechanism relating to dopamine. As with other endocrine control mechanisms, the final hormone increase suppresses secretory hormones and, if this causes an increase in inhibitory hormone, it can be expected that thyroxine will stimulate dopamine. The increase in dopamine plays a role in mechanisms relating to nausea and vomiting.^16^ Although the effects of dopamine on TSH and TRH have been researched, the effects of thyroid hormones on dopamine have not been researched. This is a significant deficiency and there is therefore a need for new research on this topic.

Some studies investigating the effect of high HCG titers on TSH and fT4 levels have shown that when the HCG titer increases above 200,000 IU/l, the probability of incidence of TSH suppression (TSH < 0.2 IU/l) is 67%, while at titers above 400,000 IU/l, TSH suppression reaches 100%.^17^^,^^18^^,^^19^ In our study, contrary to the data in the literature, there were euthyroid patients with even the highest HCG values. Among the 30 patients with HCG values above 200,000 uIU/l, 17 (70%) began treatment for clear hyperthyroidism, while among the 20 patients with HCG above 400,000 uIU/l, 15 (71%) began treatment. There are some studies with results similar to those of our research.^20^ In this group of patients who were euthyroid despite high HCG levels, we believe that the stimulating effect of HCG on the thyroid gland may have led to development of resistance at receptor levels for an unknown reason. However, there is a need for more immunological research and animal experiments to support our hypothesis.

Since the patients with complete hydatidiform mole were older, with more pregnancies and larger thyroid volumes, we think that HCG stimulation may have caused synthesis and release of more hormones. There is a need for randomized prospective studies on this topic, with more patients.

Hydatidiform mole pregnancies stimulate the thyroid not only through higher HCG levels but also through increasing estrogen concentrations.^21^ Thus, the thyroid volume increases with increasing numbers of pregnancies. Moreover, both the high estrogen concentrations in pregnancy and the increasing iodine requirements may have a stimulatory effect on growth of the thyroid.

Some difficulty in perioperative monitoring of gestational trophoblastic diseases exists. These diseases are frequently complicated by hyperthyroidism. Uncontrolled hyperthyroidism may transform into a thyroid crisis or cause serious arrhythmia during the perioperative period.^22^^-^^23^ Gestational trophoblastic neoplasia is one of the most rapidly metastasizing tumors. The first-choice medication for thyroid crises, which are a complication of hyperthyroidism requiring emergency treatment, is thionamides.^24^ In our study, none of the patients presented any thyroid crises, and so we used monotherapy consisting of PTU. The postoperative decline in HCG levels was most commonly seen in patients receiving PTU therapy.^25^

## CONCLUSION

The severity of thyroid disease in hydatidiform mole patients increases according to age, parity, beta-HCG level and mole size. Determination of thyroid volume may help in estimating the severity of thyroid disease. However, there is a need for prospective multicenter studies on this topic, with higher numbers of patients and closer patient monitoring.
